# Movement of Mandibular Incisors in Patients With Skeletal Class III During the Surgical Orthodontic Treatment

**DOI:** 10.7759/cureus.77588

**Published:** 2025-01-17

**Authors:** Yuki Iijima, Chie Tachiki, Dai Ariizumi, Masae Yamamoto, Takashi Takaki, Akira Watanabe, Akira Katakura, Yasushi Nishii

**Affiliations:** 1 Department of Orthodontics, Tokyo Dental College, Tokyo, JPN; 2 Department of Oral Pathobiological Science and Surgery, Tokyo Dental College, Tokyo, JPN; 3 Department of Oral and Maxillofacial Surgery, Tokyo Dental College, Tokyo, JPN

**Keywords:** lower incisors, mandibular symphysis, orthodontic decompensation, orthognathic surgery, skeletal class iii

## Abstract

Introduction: This study investigated changes in the morphology of the mandibular symphysis and the movement of the mandibular incisor over time during surgical orthodontic treatment for skeletal class Ⅲ.

Methods: Twenty-two patients with skeletal class Ⅲ malocclusion treated with combined orthodontic treatment, and orthognathic surgery were included. Multidetector computed tomography (CT) and lateral cephalograms were obtained from these patients to assess changes in mandibular incisor inclination, position, labial and lingual bone area, alveolar bone thickness at the mandibular central incisors and canines, and the morphological evaluation of the mandibular symphysis.

Results: The lower alveolar bone thickness at the incisors showed a significant increase, whereas the lingual alveolar bone area significantly decreased. Additionally, the incisor mandibular plane angle (IMPA) significantly increased. Labial tipping of the mandibular incisors during presurgical orthodontic treatment resulted in the root apices moving closer to the lingual cortical bone. During postsurgical orthodontic treatment, the position of the incisor root apices remained stable, while the incisor crowns underwent lingual movement.

Conclusion: Mandibular incisors incline labially during presurgical orthodontics accompanied by the lingual inclination of the mandibular symphysis. These findings highlight the dynamic changes in alveolar bone and root position during orthognathic surgical treatment.

## Introduction

When planning orthodontic treatment, identifying the position of the mandibular incisor within the mandibular symphysis is essential for establishing the treatment strategy. In cases of skeletal class III, dental compensation due to disharmony between the maxilla and mandible often results in labial inclination of the maxillary incisor and lingual inclination of the mandibular incisor. Treatment of skeletal class III includes either orthodontic treatment alone or surgical orthodontic treatment. Eslami et al. highlighted the utility of the Holdaway H angle and Wits appraisal in treatment planning and concluded that surgical orthodontic treatment is indicated when the Wits appraisal is smaller than -5.8 mm [1]. For orthodontic treatment alone, camouflage treatment is performed using class III elastics or distalization of the mandibular teeth. Surgical orthodontic treatment consists of presurgical orthodontic treatment, orthognathic surgery, and postoperative orthodontic treatment. Presurgical orthodontic treatment aims to stabilize the postsurgical occlusion by relieving crowding, correcting dental decompensation, and harmonizing the maxillary and mandibular arch forms. For skeletal class III cases, achieving dental decompensation during presurgical orthodontic treatment is a primary goal. This involves lingual movement of the maxillary incisor and labial movement of the mandibular incisor. However, in cases with a thin mandibular symphysis, maintaining the root apices within the boundaries of the thin bone is crucial to successful orthodontic outcomes.

Changes in alveolar bone thickness in the mandibular anterior region have been associated with root movement during presurgical decompensation involving labial movement of the mandibular anterior teeth [2]. Meanwhile, in cases where the labial alveolar bone is thin, labial movement of the mandibular incisor during decompensation may result in gingival recession. Choi et al. reported that presurgical labial movement of the mandibular incisors leads to regression of the labial alveolar bone and a reduction in the width of the attached gingiva [3]. For cases with thin labial alveolar bone, corticotomy is sometimes performed concurrently. Ahn et al. reported that corticotomy can augment the labial alveolar bone of the mandibular incisor and help preserve periodontal tissues [4]. Additionally, corticotomy of the mandibular anterior region can lead to the mandibular symphysis to incline labially along with the labial inclination of the mandibular anterior teeth [5]. While studies have examined the axial inclination of the mandibular incisor and the morphology of the mandibular symphysis during presurgical orthodontic treatment for surgical orthodontics [6,7], few have investigated the movement pattern of the mandibular anterior teeth within the symphysis over time. This study investigated changes in the morphology of the mandibular symphysis and the movement of the mandibular incisor over time during surgical orthodontic treatment for skeletal class III.

## Materials and methods

This retrospective study included 22 patients (5 male, 17 female; mean age at initial consultation: 23 years 7 months ± 8 years 0 months) diagnosed with skeletal class III, who were treated at the Department of Orthodontics of Tokyo Dental College Chiba Dental Center between January 1998 and January 2018. All patients had completed surgical orthodontic treatment and achieved stable occlusion two years post-retention. Patients with congenital jaw or oral cavity abnormalities, a history of trauma, missing teeth (excluding third molars), prior orthodontic treatment, or prosthetic treatment involving the mandibular anterior region were excluded. (1) The inclusion criteria for the study patients based on lateral cephalometric radiograph analysis at the pretreatment were as follows: (i) the Wits appraisal had to show a value smaller than -6 mm; (ii) the lateral deviation of Menton relative to the midsagittal plane had to be within ±3 mm. (2) Additionally, model analysis criteria stipulated as follows: (i) two years post-retention, the overjet and overbite had to remain within the range of +1.5 mm to +4 mm; (ii) there is almost no difference between the left and right mandibular central incisors and canines. Cases meeting both selection requirements (1) and (2) were analyzed. Table 1 shows the demographic data of the patients.

**Table 1:** Demographic date of the patients SNA: Sella-Nasion-Point A angle; SNB: Sella-Nasion-Point B angle; ANB: Point A-Nasion-Point B angle; IMPA: incisor mandibular plane angle; ALD: arch length discrepancy

Characteristics	Values
Age	23 years 7 months ± 8 years 0 months
Angle	Class Ⅲ
Surgery	2 jaw
Wits appraisal (mm)	-11.35 ± 3.29
SNA (°)	77.75 ± 2.7
SNB (°)	79.95 ± 3.9
ANB (°)	−2.1 ± 3.2
IMPA (°)	81.7 ± 5.7
Upper ALD (mm)	-5.0 ± 3.66
Lower ALD (mm)	-4.8 ± 2.58
Curve of Spee (mm)	-1.1 ± 1.08

The Malocclusion classification of the angle at the pretreatment was class III for all patients. During presurgical orthodontic treatment, no extractions were performed in the mandible. In the maxilla, 16 patients did not undergo extraction, while six patients underwent extraction of the maxillary left and right first premolars. The arch length discrepancy (ALD) was -5.0 ± 3.66 mm in the maxilla and -4.8 ± 2.58 mm in the mandible. Treatment was performed by two orthodontists with over 10 years of experience in surgical orthodontics and three oral surgeons with over 20 years of experience in orthognathic surgery. A 0.022 × 0.028 slot straight-wire appliance was used for the surgical orthodontic treatment. During presurgical orthodontic treatment, levelling was performed using Ni-Ti wires, and the wire size was gradually increased to 0.019 × 0.025 Ni-Ti wires. The final archwire used before surgery was a 0.019 × 0.025 stainless steel wire, which was employed to align the arch form. The duration of presurgical orthodontic treatment was 1 year 7 months ± 6 months. This study was approved by the Ethics Committee of Tokyo Dental College (Approval Number 870).

**Figure 1:**
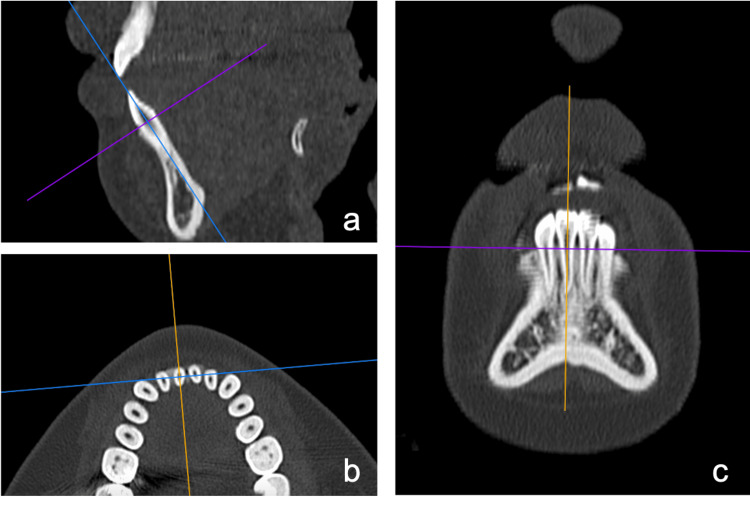
The measurement section of the CT image (a) Sagittal view of lower central incisor; (b) coronal section; (c) frontal section. Image Credits: Yuki iijima

Measurements were made using multidetector computed tomography (CT) and lateral cephalometric radiographs taken at the following time points: pretreatment (T1), end of presurgical orthodontic treatment (T2), one month after orthognathic surgery (T3), and two years after orthognathic surgery (T4). CT imaging was performed using the Somatom Plus4 VolumeZoom (Siemens, Erlangen, Germany). The imaging conditions were set as follows: tube voltage of 120 kV, tube current of 100 mA, and a field of view (FOV) of 230 mm. All imaging was performed under the same conditions. The CT data were converted to DICOM format, and the mandibular incisor region was selected from the coronal section. The position and angle of the teeth were defined, and the long axis of the teeth, as observed in the coronal section, was adjusted vertically in the frontal view. A sagittal section of the mandibular central incisor was then created. Figure 1 shows the measurement section of the CT image.

**Figure 2:**
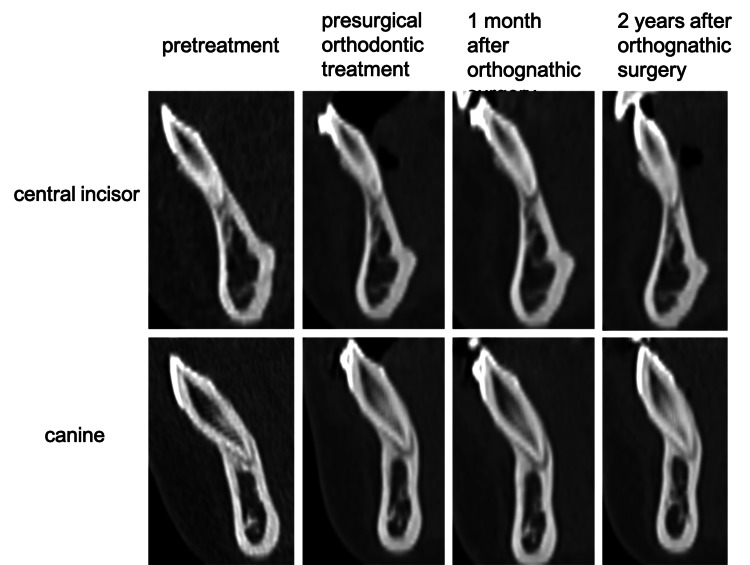
CT imaging of the lower central incisor and canine Image Credits: Yuki Iijima

Measurements were performed using the image analysis software, Simplant OMS Pro (Materialize Denta Co., Ltd., Leuven, Belgium). To evaluate the thickness of the labial bone of the mandibular central incisors and canines on the CT, the sagittal section along the long axis of the mandibular incisor and canine was used. Figure 2 shows the CT imaging of the lower central incisor and canine.

**Figure 3:**
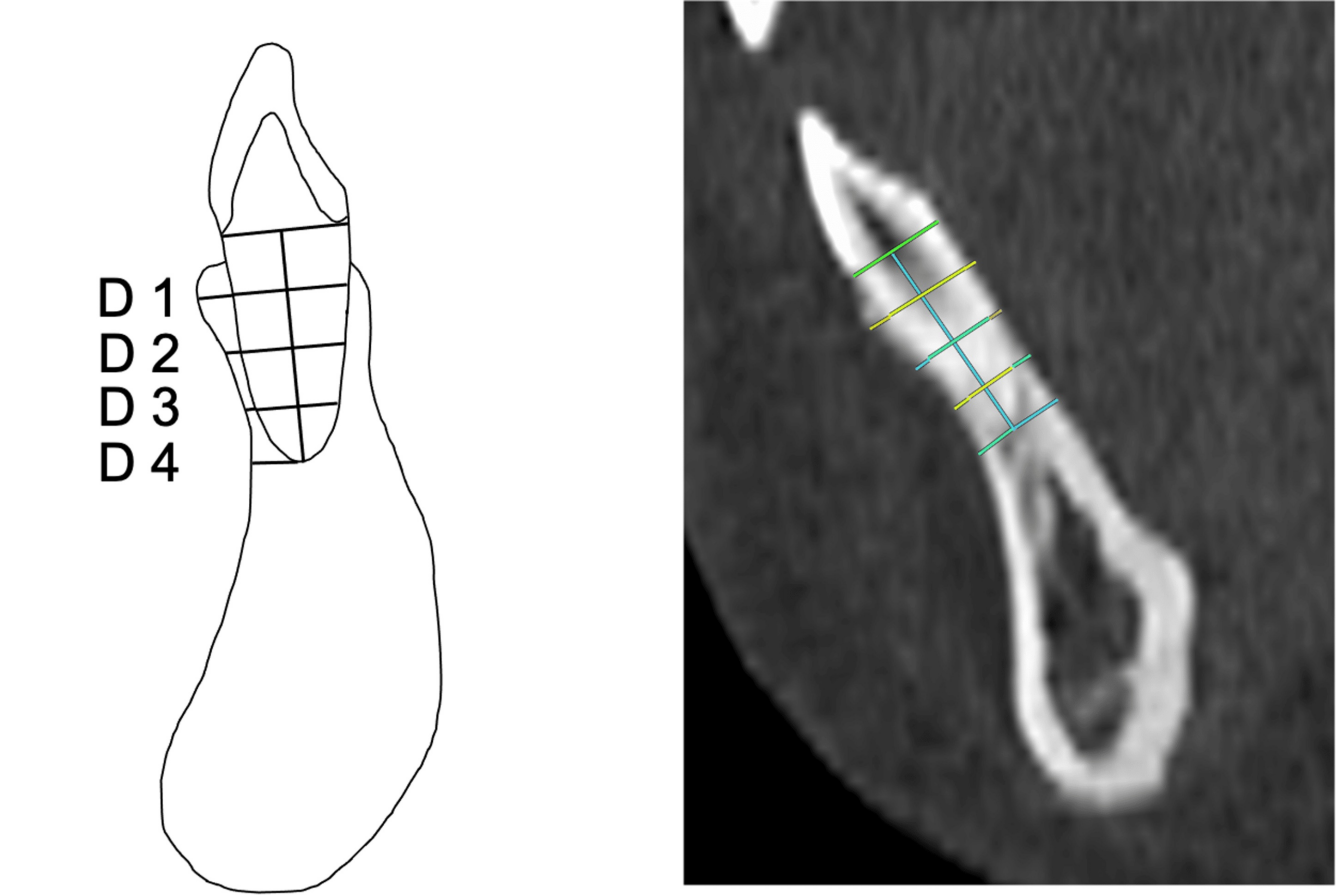
CT image and illustrations of reference points, lines and measurement variables The alveolar bone thickness is measured between the surface of the labial cortical plates at the levels of D1, D2, D3, and D4. A line from the anatomical cervical line to the root apex is divided into four segments, with measurements taken at the cervical area (D1), and progressively at D2, D3, and D4. Image Credits: Yuki Iijima

The distance (D) between the root and the labial alveolar bone surface was measured. A line from the anatomical cervical line to the root apex was divided into four segments, with measurements taken from the cervical area to D1, D2, D3, and D4, respectively. Figure 3 shows the CT image and the illustrations of the measurement variables.

**Figure 4:**
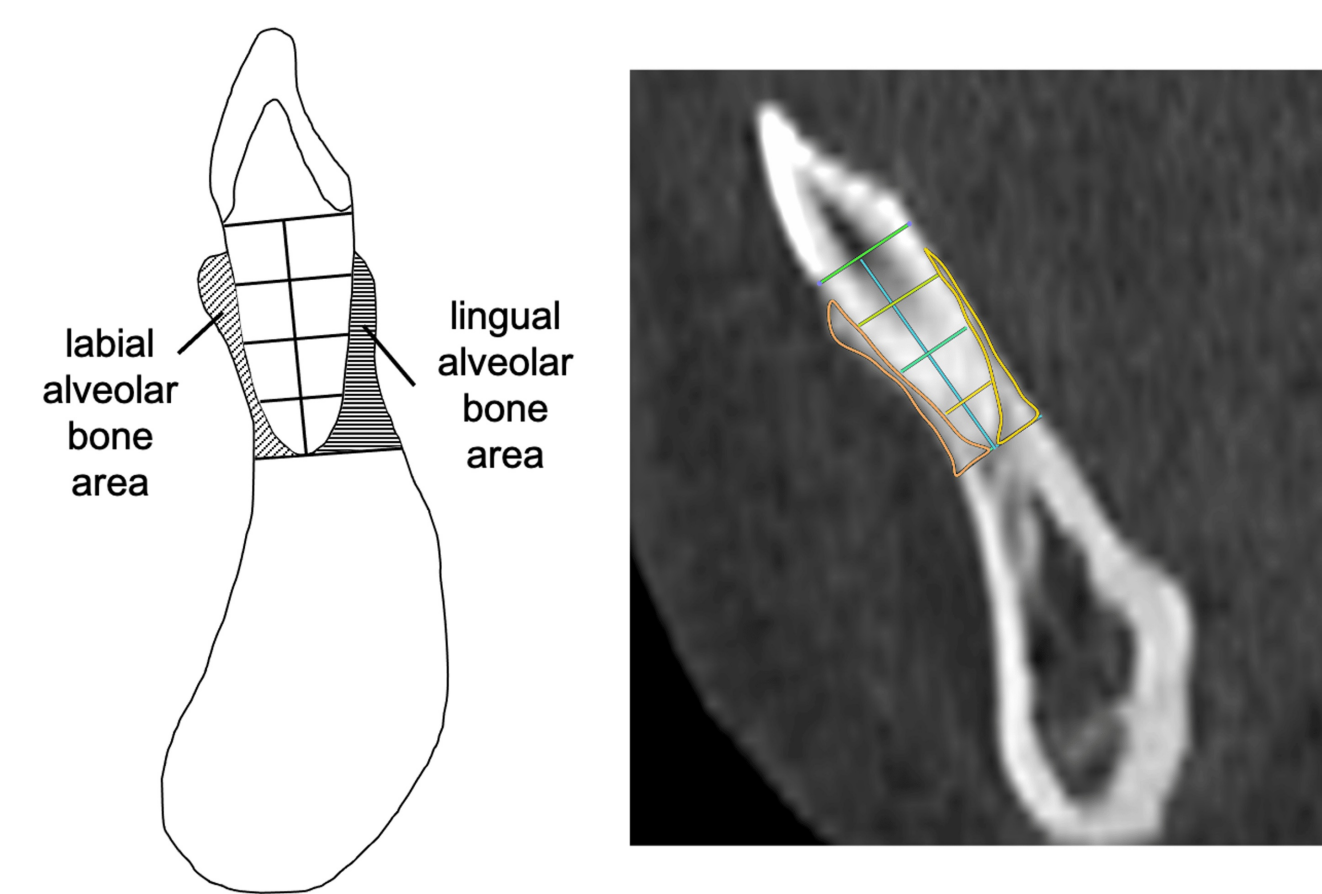
The measurements of the alveolar bone areas around the root of the mandibular central incisor Image Credits: Yuki Iijima

Additionally, the labial and lingual alveolar bone areas around the root of the mandibular central incisor were measured on the sagittal section along the long axis of the tooth. Figure 4 shows the CT image and the illustrations of measurement variables.

**Figure 5:**
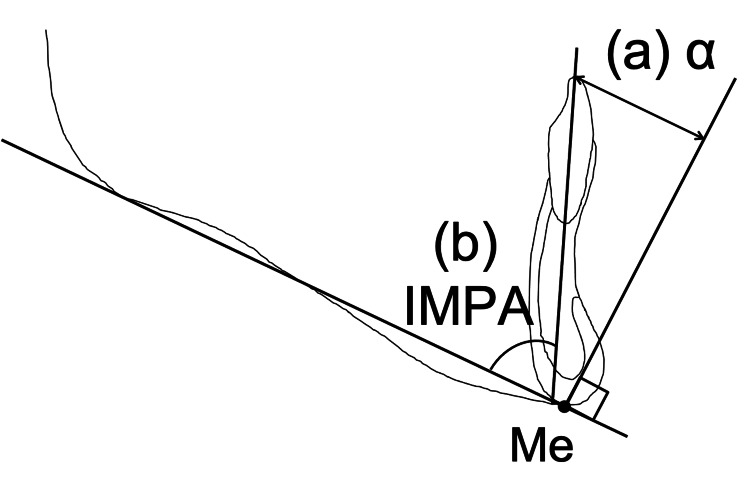
Cephalometric measurements (a) Horizontal distance of the mandibular incisor tip to the perpendicular line to the mandibular plane at Me. (b) IMPA (incisor mandibular plane angle) Image Credits: Yuki Iijima

In the lateral cephalometric radiographs, the incisor mandibular plane angle (IMPA) was measured in relation to the mandibular plane to evaluate the axial inclination of the mandibular central incisor. The distance (α) between the perpendicular line from the Me point to the mandibular plane and the incisal edge of the mandibular central incisor was measured to assess the horizontal position of the incisal edge of the mandibular central incisor. Figure 5 shows the cephalometric measurements.

**Figure 6:**
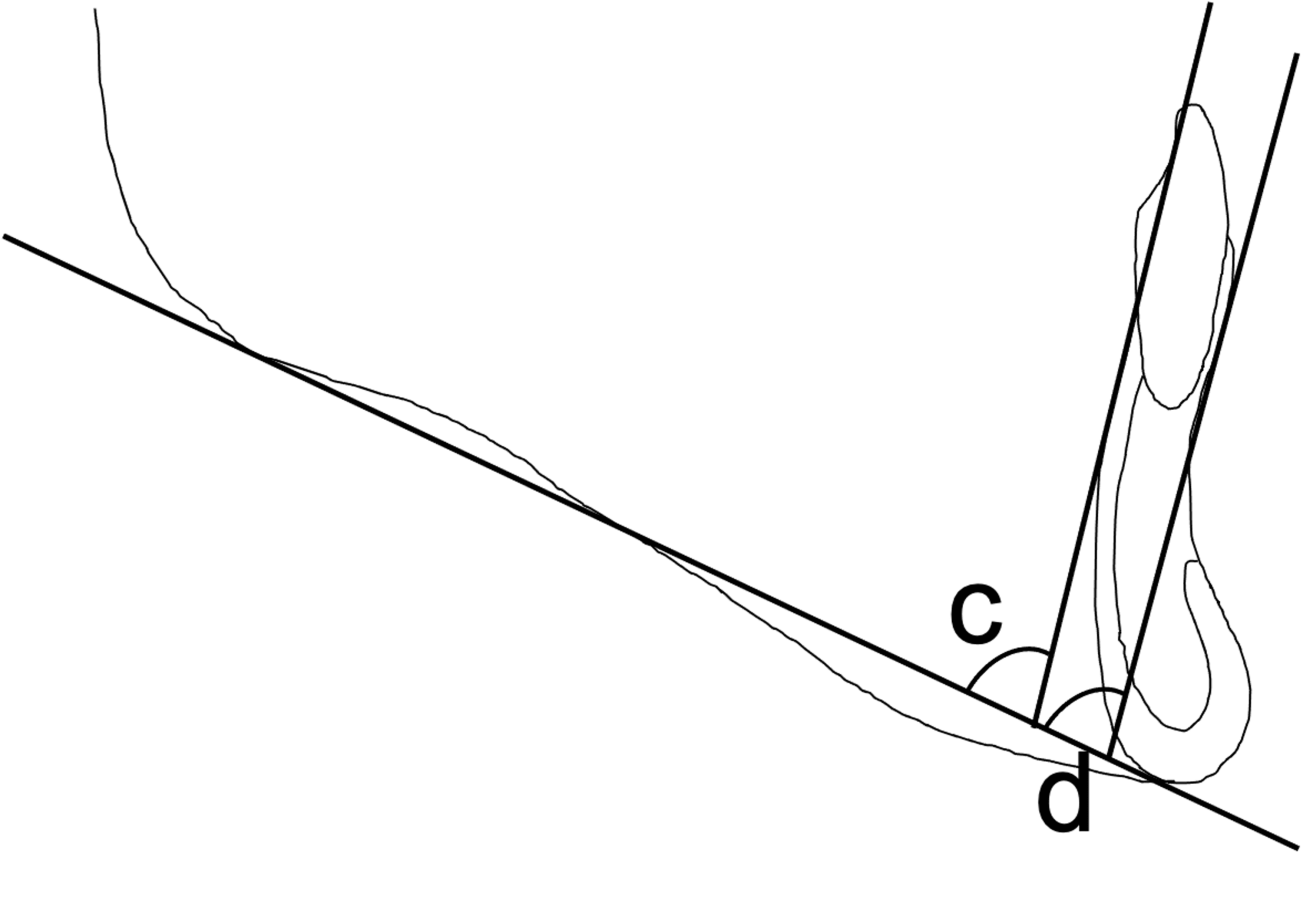
Cephalometric measurements The angle formed between the tangent to the upper part of the mandibular symphysis and the mandibular plane was measured. (c) Lingual alveolar bone angle. (d) Labial alveolar bone angle. Image Credits: Yuki Iijima

For the morphological evaluation of the mandibular symphysis, the angle formed between the tangent to the upper part of the mandibular symphysis and the mandibular plane was measured as the labial and lingual alveolar bone angles. Figure 6 shows the cephalometric measurements.

All measurements were performed by a single examiner. To assess measurement error, three measurements were taken at a two-week interval for 10 patients, and inter-rater reliability was tested using the intraclass correlation coefficient (ICC). The ICC (1,3) was 0.988, indicating that the measurements were reproducible. The sample size was calculated using G*Power software (Heinrich-Heine-Universität Düsseldorf, Düsseldorf, Germany), and 22 patients were included in the study.

The analysis of changes from T1 to T4 was performed using IBM SPSS Statistics for Windows (Version 19.0. Armonk, NY: IBM Corp.). Statistical processing was conducted using the Friedman test and the Wilcoxon signed-rank test. The statistical significance level was set at P < 0.05.

## Results

Thickness of the labial alveolar bone of the mandibular central incisor

**Figure 7:**
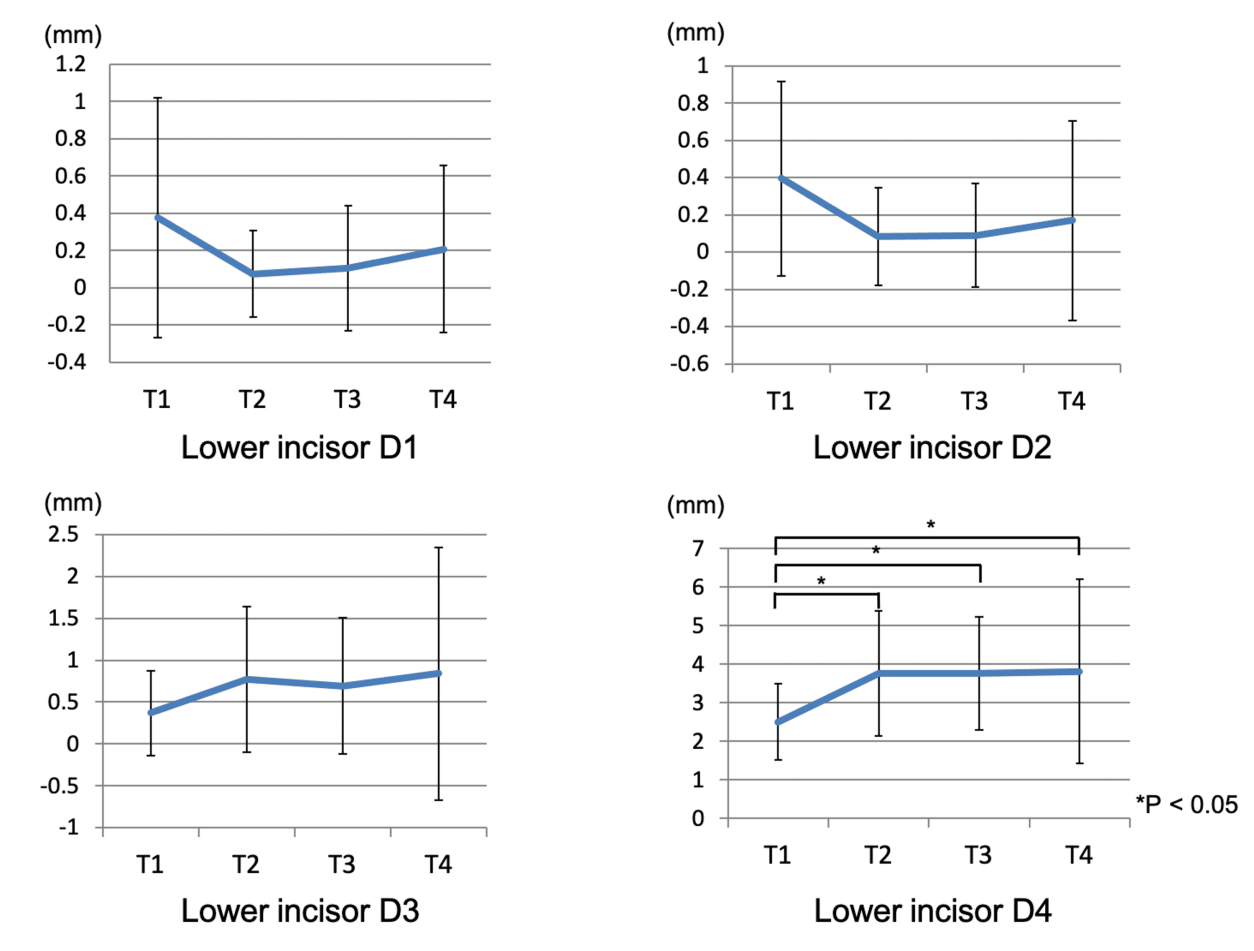
The change of the alveolar bone thickness at the each level of the mandibular incisor T1: pretreatment; T2: end of presurgical orthodontic treatment; T3: one month after orthognathic surgery; T4: two years after orthognathic surgery. * statistically significant.

CT measurements revealed significant differences at D1 (cervical side) and D4 (apical side) (p < 0.05), but no significant differences were found at D2 and D3 (central part of the root). For D4 (apical side), the measurements were as follows: 2.49 ± 0.99 mm at T1, 3.76 ± 1.61 mm at T2, 3.76 ± 1.46 mm at T3, and 3.82 ± 2.38 mm at T4. Significant differences were observed between T1 and T2, T1 and T3, and T1 and T4 (p < 0.05), with measurements at T2, T3, T4 being significantly larger compared to T1. Figure 7 shows the change in the labial alveolar bone thickness at each level.

Alveolar bone area around the mandibular central incisor root

**Figure 8:**
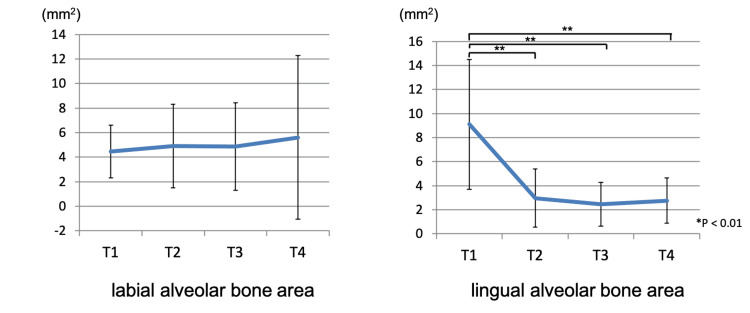
The change of the alveolar bone area around the mandibular incisor root T1: pretreatment; T2: end of presurgical orthodontic treatment; T3: one month after orthognathic surgery; T4: two years after orthognathic surgery. ** statistically significant.

No significant differences were observed in the labial alveolar bone area. However, significant differences were noted between T1-T2, T1-T3, and T1-T4 (p < 0.01) in the lingual alveolar bone area (9.10 ± 5.40 mm² at T1, 2.96 ± 2.42 mm² at T2, 2.46 ± 1.82 mm² at T3, and 2.74 ± 1.89 mm² at T4). A significant reduction in lingual alveolar bone area was observed at T2, T3, and T4 compared to T1. Figure 8 shows the change in the alveolar bone area around the mandibular central incisor root.

Changes in the dental axis of the mandibular central incisor

**Figure 9:**
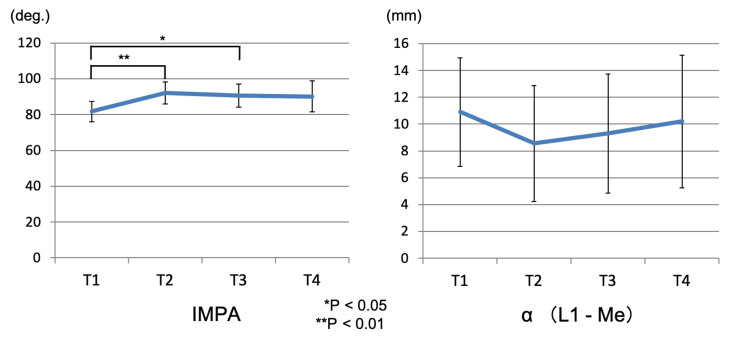
The change in the dental axis of the mandibular incisor IMPA: incisor mandibular plane angle; α (L1-Me): the distance between the perpendicular line from the Me point to the mandibular plane and the incisal edge of the mandibular incisor; T1: pretreatment; T2: end of presurgical orthodontic treatment; T3: one month after orthognathic surgery; T4: two years after orthognathic surgery. *, ** statistically significant.

No significant differences were observed in the position of the mandibular central incisor incisal edge (α), as measured by lateral cephalometric radiography. The IMPA, which indicates the inclination of the mandibular central incisor relative to the mandibular plane, was 81.7 ± 5.68° at T1, 92.05 ± 6.16° at T2, 90.6 ± 6.36° at T3, and 90.1 ± 8.67° at T4. Significant differences were found between T1-T2 (p < 0.01) and T1-T3 (p < 0.05), showing a larger labial inclination of the mandibular incisors at T2 and T3 compared to T1. Figure 9 shows the change in the dental axis of the mandibular incisor.

Mandibular canines

**Figure 10:**
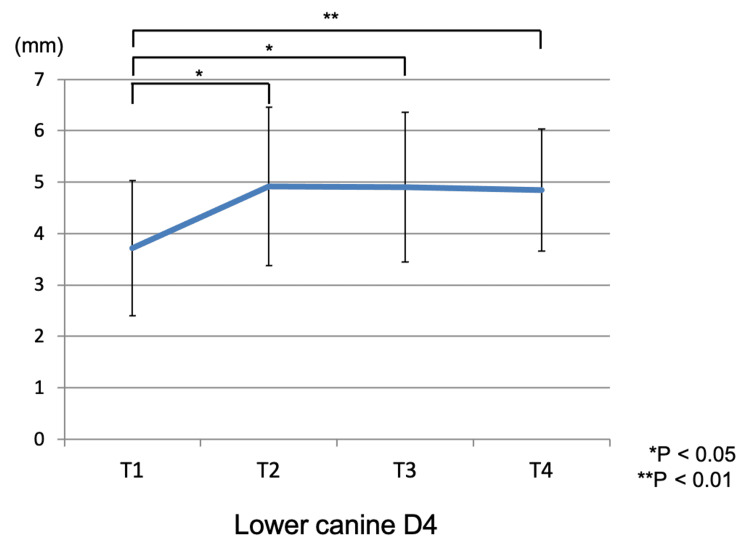
The change of the alveolar bone thickness in the mandibular canine T1: pretreatment; T2: end of presurgical orthodontic treatment; T3: one month after orthognathic surgery; T4: two years after orthognathic surgery. *, ** statistically significant.

CT measurements for D4 revealed the following values: 3.7 ± 1.31 mm at T1, 4.92 ± 1.55 mm at T2, 4.90 ± 1.46 mm at T3, and 4.85 ± 1.16 mm at T4. Significant differences were found between T1-T2 (p < 0.05), T1-T3 (p < 0.05), and T1-T4 (p < 0.01). Measurements at T2, T3, and T4 were significantly larger than T1. Figure 10 shows the change of the alveolar bone thickness at D4 of the mandibular canine.

Changes in mandibular symphysis morphology

**Figure 11:**
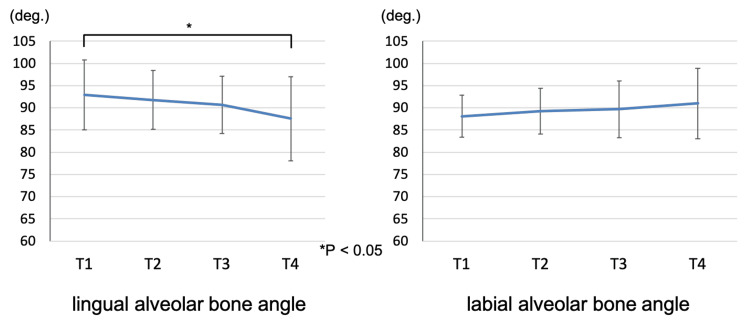
The change in the mandibular symphysis morphology T1: pretreatment; T2: end of presurgical orthodontic treatment; T3: one month after orthognathic surgery; T4: two years after orthognathic surgery. * statistically significant.

The lingual alveolar bone angle of the mandibular symphysis decreased significantly from 92.9 ± 7.9° at T1 to 87.6 ± 9.5° at T4 as measured by lateral cephalometric radiography (p < 0.05). The labial alveolar bone angle of the mandibular symphysis was 88.1 ± 4.7° at T1 and 91.0 ± 7.9° at T4, with no significant difference observed. Figure 11 shows the change in mandibular symphysis morphology.

## Discussion

This study evaluated the movement of the mandibular incisor in the mandibular symphysis using lateral cephalometric radiographs and CT. While the evaluation of the mandibular incisor and the mandibular symphysis has traditionally relied on lateral cephalograms, recent studies have increasingly utilized cone-beam computed tomography (CBCT). Compared to two-dimensional cephalograms, CBCT offers more precise information for evaluating the alveolar bone [8,9]. However, in this study, CT images obtained for orthognathic surgery planning and postoperative evaluations were used to assess the position of tooth roots within the mandibular symphysis in order to avoid unnecessary radiation exposure.

Yamada et al. used CT to evaluate the mandibular incisors at the mandibular symphysis and reported that in adults with an untreated skeletal mandibular protrusion, the lingual inclination of mandibular incisors caused the surrounding alveolar bone to incline lingually and become thinner, with the spices moving closer to the labial cortical bone [10]. Similarly, in this study, the mandibular incisors were inclined lingually at the pretreatment and the apices were close to the labial alveolar bone.

Presurgical orthodontic treatment for skeletal mandibular protrusion induces labial inclination of the mandibular incisor as part of dental decompensation [11]. In this study, a significant difference in the IMPA was observed between T1 and T2 during presurgical orthodontic treatment, indicating labial inclination of the mandibular central incisors. This study focused on patients undergoing surgical orthodontic treatment, and the evaluation of the dental axis and horizontal position of the mandibular incisor was based on the mandibular plane, ensuring consistency unaffected by treatment.

Regarding the movement of the mandibular central incisor root within the mandibular symphysis during presurgical orthodontic treatment, no significant difference was observed in the horizontal position of the crown at α; however, a significant difference was noted at the apical D4. This suggests that the movement of the tooth was more influenced by the apical movement toward the lingual side than by the crown’s labial movement. As for root resorption, no significant change in root length was observed in this study. The absence of significant differences in parameters between the presurgical and postsurgical stages is likely due to the short duration of measurement periods and limited tooth movement immediately before and after orthognathic surgery. Significant differences in D4 and lingual bone volume were observed between the initial visit and two years postsurgery, whereas no significant differences occurred during postsurgical orthodontic treatment. This indicates minimal apical movement during the postsurgical phase. Vertical facial pattern is an important factor in mandibular symphysis morphology and lower incisor positioning. The morphology of the mandibular symphysis is known to differ between short- and long-faced individuals, with long-faced individuals having thinner mandibular symphyses, which impose greater restrictions on the movement of the mandibular incisor [12]. In this study, long-faced patients were 4, average-faced patients were 11, and short-faced patients were 7. Additionally, since the inclination of the mandibular incisor affects alveolar bone volume, care must be taken to ensure tooth movement remains within the alveolar bone [13]. The extrusion of the mandibular incisor affects the labial inclination of the mandibular incisor during presurgical treatment. Sendyk et al. conducted a three-dimensional evaluation of the changes in mandibular symphysis morphology and reported that the buccolingual movement of the mandibular incisor is correlated with the inclination of the mandibular symphysis and the B-point, but not with the thickness of the mandibular symphysis [14]. In this study, the postsurgical lingual alveolar bone angle in the mandibular symphysis was smaller than that measured at the initial consultation, and the mandibular symphysis also moved lingually.

No significant difference was observed in α (the horizontal position of the mandibular central incisor at the incisal edge), there was a tendency for an increase from T2 to T4, suggesting that the crown of the mandibular central incisor may have moved lingually. This could be attributed to the influence of class III elastics used during postsurgical orthodontic treatment to stabilize jaw position.

Due to measurement challenges in lateral cephalometric radiographs, mandibular canine measurements were only performed using CT. The thickness of the labial alveolar bone at the apical area, D4, of the mandibular canine was similar to that of the central incisor. This suggests that during presurgical orthodontic treatment, mandibular canines inclined labially, with primary movement occurring at the apical region due to lingual movement.

A limitation of this study is that evaluation was performed using CT, making it difficult to assess the alveolar bone in more detail compared to CBCT. In addition, since the measurement point is set along the tooth axis of the mandibular incisor, the measurements may change if root resorption occurs. No significant change in root length was observed in this study and unlikely to have affected the results. All subjects were at an age when maxillofacial growth was nearly complete, and it is unlikely that growth changes in the jaw or alveolar bone would have affected the results. However, vertical facial patterns include long-faced and short-faced, and additional studies are needed to extend the conclusions to facial patterns.

## Conclusions

In surgical orthodontic treatment for skeletal class III, dental compensation due to disharmony between the maxilla and mandible often results in lingual inclination of the mandibular incisor. Mandibular incisors incline labially during presurgical orthodontic treatment, but this is due to lingual movement of the root apex rather than labial movement of the crowns. Postsurgical orthodontic treatment showed no apical movement but suggested lingual movement of the crown. At that time, the mandibular symphysis moved lingually. In cases with thin mandibular symphyses, special care is needed to avoid excessive labial movement of mandibular incisors.
